# Decrypting plant tissues: From bulk to cell-type transcriptional profiles

**DOI:** 10.1093/plphys/kiae190

**Published:** 2024-04-02

**Authors:** Sebastián R Moreno

**Affiliations:** Assistant Features Editor, Plant Physiology, American Society of Plant Biologists; Sainsbury Laboratory, University of Cambridge, Bateman Street, Cambridge CB2 1LR, UK

Recent advancements in single-cell RNA-sequencing (scRNA-seq) are powerful tools to decipher cell-type transcriptional profiles and differentiation dynamics in plant biology. Studies employing scRNA-seq have provided insights into complex developmental processes such as root differentiation (Shahan et al. 2022), leaf development (Procko et al. 2022), and stomatal differentiation (Lopez-Anido et al. 2021), among others.

Despite the invaluable insights provided by single-cell transcriptomic approaches, they are inaccessible for many researchers due to the number of datasets required, particularly in time series experiments. Although high-resolution RNA-seq experiments can demonstrate gene expression changes over time, most of these datasets have been collected from isolated tissues, lacking the nuance that cell type–specific profiles offer. To address this limitation, computational techniques leveraging deconvolution methods have been developed, enabling the extraction of cell type–specific gene expression profiles from bulk RNA-seq data without the need for physical isolation of single cells (Newman et al. 2019; Sutton et al. 2022).

In a pioneering study published in this issue of *Plant Physiology*, Vong et al. (2024) explored the feasibility of the computational framework CIBERSORTx to infer cell-type expression from bulk RNA-seq time series in plants (Newman et al. 2019). Given that CIBERSORTx or other scRNA-seq deconvolution methods have never been tested in *Arabidopsis thaliana*, the authors first evaluated the accuracy of this pipeline in predicting cell-type proportions in bulk RNA-seq samples by training CIBERSORTx with the scRNA-seq atlas of mature leaves (Fig. A) (Procko et al. 2022). By using 75% of cells to train CIBERSORTx and the rest to simulate 3 replicates of bulk RNA-seq, the authors observed that this deconvolution method accurately identified all cell types from the original clustering approach: 3 mesophyll cell clusters, epidermis, vasculature-related clusters, and many others. To be more precise with CIBERSORTx output, the authors used the concept of cell-type “activity” instead cell-type gene expression.

**Figure.**
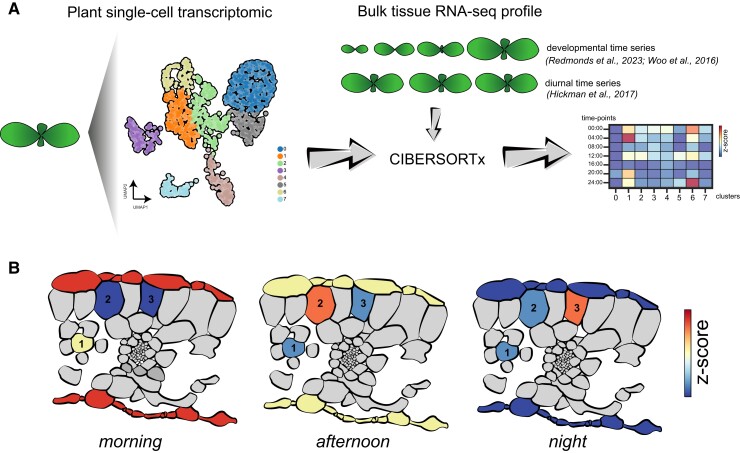
CIBERSORTx reconstruct cell-type transcriptional profiles from bulk RNA-seq in plants. **A)** CIBERSORTx workflow used in Vong et al. (2024), illustrating the integration of published single-cell RNA-seq datasets from leaves to derive cell-type profiles from time-series bulk RNA-seq data. **B)** Graphical abstract depicting orthogonal view of leaves highlighting cell type–specific expression activity for epidermis and the three mesophyll cell clusters identified from diurnal time series RNA-seq datasets using the CIBERSORTx approach and Procko et al.(2022) single-cell datasets.

Vong and colleagues then used a published time-series bulk RNA-seq dataset profiling Arabidopsis leaves from 4 to 30 days of age (Fig. A) (Woo et al. 2016). Using the CIBERSORTx and the signature matrices generated, Vong and colleagues observed a decrease in epidermal activity, increase in vascular activity, and enhanced phloem parenchyma transcriptional activity, which is consistent with previous reports in senescing leaves. Additionally, the authors analyzed a previously published leaf time-series dataset spanning different diurnal time scales (Hickman et al 2017). The CIBERSORTx pipeline effectively captured differences in the transcriptional activity of different cell types along the diurnal time series experiments. For instance, epidermal activity peaked in the morning (Fig. B), while vascular transcriptional activity was enhanced in the afternoon. Interestingly, the 3 identified mesophyll cell types displayed time-dependent activity (Fig. B). Thus, the authors were able to capture cell type–specific activity not previously observed in the original bulk RNA-seq.

Subsequently, the authors investigated the expression of light-responsive genes in the different deconvoluted cell types. Since group 1 mesophyll cells are more active in the morning, the authors tested whether this group of cells exhibited more light-responsive genes activated at night. Remarkably, they confirmed that light-responsive genes are induced at night in the group 1 mesophyll, along with another group of light-responsive genes expressed in the stress mesophyll cluster.

Finally, Vong and colleagues examined whether the CIBERSORTx method could capture cell type–specific responses from bulk RNA-seq obtained the during bolting transition. Leveraging a public dataset (Redmond et al. 2023), they observed heightened transcriptional activity in cell types such as mesophyll group 1, mesophyll group 2, vasculature, and companion cell types after bolting, while other cell identities such as epidermis and mesophyll group 3 showed a peak of transcriptional activity before bolting. Gene Ontology analysis revealed that increased transcriptional activity during bolting in mesophyll group 2 is associated with ATP, GTP, NAD+ binding, and endocytosis regulation. By contrast, decreased epidermal transcriptional activity during bolting was enriched in genes associated with chloroplast, photosynthesis, and amino acid metabolism, among others. Thus, the authors identified transcriptional activities during a developmental shift such as bolting and identified genes and biological processes that could potentially explain bolting transition at cell-type specific resolution.

In conclusion, this study confirms that time-lapse bulk RNA-seq datasets can be used to obtain dynamic cell type–specific transcriptional profiles by leveraging single-cell transcriptomics. As a logical progression, future research comparing available time-series scRNA-seq data with deconvoluted bulk RNA-seq time-series will refine the resolution of information derived from bulk RNA-seq datasets. The findings in this study demonstrate the feasibility of employing CIBERSORTx computational approach in plant time-series experiments. Hence, this work highlights a promising avenue for employing bulk RNA-seq datasets as a means to provide cell-type specific profiles and greater access to these data for the whole scientific plant community.
